# Psychosocial Outcomes with the Omnipod® 5 Automated Insulin Delivery System in Children and Adolescents with Type 1 Diabetes and Their Caregivers

**DOI:** 10.1155/2023/8867625

**Published:** 2023-06-29

**Authors:** Korey K. Hood, William H. Polonsky, Sarah A. MacLeish, Carol J. Levy, Gregory P. Forlenza, Amy B. Criego, Bruce A. Buckingham, Bruce W. Bode, David W. Hansen, Jennifer L. Sherr, Sue A. Brown, Daniel J. DeSalvo, Sanjeev N. Mehta, Lori M. Laffel, Anuj Bhargava, Lauren M. Huyett, Todd E. Vienneau, Trang T. Ly

**Affiliations:** ^1^Department of Pediatrics, Psychiatry & Behavioral Sciences, Stanford Diabetes Research Center, Stanford University School of Medicine, Stanford University, Stanford, CA, USA; ^2^University of California San Diego, San Diego, CA, USA; ^3^Behavioral Diabetes Institute, San Diego, CA, USA; ^4^University Hospitals Cleveland Medical Center, Rainbow Babies and Children's Hospital, Cleveland, OH, USA; ^5^Icahn School of Medicine at Mount Sinai, New York, NY, USA; ^6^Barbara Davis Center for Diabetes, University of Colorado Anschutz Medical Campus, Aurora, CO, USA; ^7^International Diabetes Center, Health Partners Institute, Minneapolis, MN, USA; ^8^Department of Pediatrics, Division of Pediatric Endocrinology, Stanford University, Stanford, CA, USA; ^9^Atlanta Diabetes Associates, Atlanta, GA, USA; ^10^Department of Pediatrics, SUNY Upstate Medical University, Syracuse, NY, USA; ^11^Department of Pediatrics, Yale School of Medicine, New Haven, CT, USA; ^12^Division of Endocrinology, Center for Diabetes Technology, University of Virginia, Charlottesville, VA, USA; ^13^Department of Pediatrics, Baylor College of Medicine, Houston, TX, USA; ^14^Joslin Diabetes Center, Harvard Medical School, Boston, MA, USA; ^15^Department of Research, Iowa Diabetes Research, West Des Moines, IA, USA; ^16^Insulet Corporation, Acton, MA, USA

## Abstract

**Objective:**

While automated insulin delivery (AID) systems aim to improve glycemic outcomes, the opportunity to improve psychosocial outcomes is also of critical importance for children and adolescents with type 1 diabetes and their caregivers. We evaluated psychosocial outcomes in these groups during a clinical trial of a tubeless AID system, the Omnipod® 5 Automated Insulin Delivery System.

**Methods:**

This single-arm, multicenter, prospective study enrolled 83 children (6.0–11.9 years) and 42 adolescents (12.0–17.9 years) with type 1 diabetes to use a tubeless AID system for 3 months. Participants and their caregivers completed age- and role-appropriate validated questionnaires to assess changes in psychosocial outcomes—diabetes distress (PAID), hypoglycemia confidence (HCS), well-being (WHO-5), sleep quality (PSQI), insulin delivery satisfaction (IDSS), and system usability (SUS)—before and after 3 months of AID system use. Associations between participant characteristics and glycemic outcomes with psychosocial measures were evaluated using linear regression analyses.

**Results:**

Improvements were found for children, adolescents, and/or their caregivers for diabetes-related distress, insulin delivery satisfaction, and system usability (all *P* < 0.05). Caregivers of children saw additional benefits of improved general well-being, confidence in managing hypoglycemia, and sleep quality (all *P* < 0.05). Regression analyses showed that improvements in psychosocial outcomes were generally independent of baseline characteristics and changes in glycemic outcomes.

**Conclusions:**

The tubeless AID system was associated with significant improvements in a number of psychosocial outcomes for children, adolescents, and their caregivers. *Trial registration*: This trial is registered with NCT04196140.

## 1. Introduction

Type 1 diabetes (T1D) is often described as a condition that affects the entire family [1, 2]. Facing a chronic medical condition at a young age is mentally taxing and can be an intimidating process for all those involved [3]. Once children or adolescents are diagnosed with T1D, caregivers are typically expected to become adept in their child's diabetes management very quickly. Automated insulin delivery (AID) systems have the potential to address multiple physiological variables that impact glycemia while reducing the burden of constant oversight of insulin dosing for optimization of glycemic control. A significant component to the success of these AID systems is the effect on children and adolescents with T1D, as well as their caregivers' overall and diabetes-related quality of life (QOL) and sleep quality [4–7]. In addition to being safe and effective, improved satisfaction with diabetes treatment and reduced burden for those affected by T1D are other important outcomes to consider for AID systems.

The Omnipod® 5 Automated Insulin Delivery System consists of a tubeless insulin pump (Pod) with a built-in algorithm that communicates directly with a continuous glucose monitor (CGM) and a compatible smartphone or handheld controller equipped with the Omnipod 5 App, described in more detail in other reports [8, 9]. Recent studies of the Omnipod 5 System have demonstrated safety, with minimal episodes of severe hypoglycemia and diabetic ketoacidosis, in addition to a decrease in hemoglobin A1c (HbA1c) of (mean ± S.D.) 0.71 ± 0.63% and 0.38 ± 0.54% and an increase in percent time in range (TIR, % 70–180 mg/dL) of 15.6 ± 11.5% and 9.3 ± 11.8% in children and adults, respectively, following 3 months of system use across people with T1D of a wide age range from 6 to 70 years [10]. Similar outcomes have also been observed in very young children aged 2 to 6 years after 3 months of Omnipod 5 use [11]. These pivotal trials supported clearance by the United States Food and Drug Administration and CE-mark for people aged 2 years or older with type 1 diabetes.

In addition to improving glycemic outcomes, it is important that AID systems improve diabetes-related QOL outcomes to support broad adoption and sustained use of these technologies. Diabetes distress and management burden affect both children and adolescents with T1D and their caregivers, and these have been associated with reduced quality of life and lower attainment of glycemic targets [12]. Several AID clinical trials have documented a positive impact on diabetes distress in this population and their caregivers [4–7]. While research with AID systems remains limited, a tubeless AID system recently demonstrated unique psychosocial benefits in adults with T1D after 3 months of system use [13]. To further assess the psychosocial benefits of a tubeless AID system during the pivotal trial of the system using investigational devices [10], we assessed changes in psychosocial measures, including diabetes distress, confidence in managing hypoglycemia, well-being, sleep quality, insulin delivery satisfaction, and perceived system usability in children (6–11.9 years) and adolescents (12–17.9 years) with T1D and their caregivers in the Omnipod 5 3-month trial.

## 2. Research Design and Methods

### 2.1. The Omnipod 5 Pivotal Trial

A single-arm, prospective, multicenter clinical study was conducted at 17 institutions in the United States. The protocol was approved by a central institutional review board and relevant local review boards. As this study took place preclearance, the U.S. Food and Drug Administration approved an investigational device exemption. Details of the study design and primary outcomes have been published previously [10]. Key inclusion criteria consisted of a point-of-care screening HbA1c <10.0% (86 mmol/mol), diagnosis with T1D for a minimum 6 months, and no history of severe hypoglycemia or diabetic ketoacidosis in the past 6 months. After participant screening, glucose sensor data were collected for two weeks while using their usual therapy (“standard therapy” (ST) phase) to later compare with the AID system phase. The study sensor was blinded during the ST phase for participants not using the Dexcom G6 CGM as part of their usual therapy regimen.

After enrollment and collection of baseline measures, participants were trained to use the investigational system, consisting of a tubeless insulin pump (Pod) with embedded automated insulin delivery algorithm (Omnipod 5, Insulet Corporation, Acton, MA), interoperable glucose sensor (Dexcom G6®, Dexcom Inc., San Diego, CA), and a smartphone application (Omnipod 5 app) on a locked-down Android phone. Additional system function details were previously published [8–10]. Participants used the system for 3 months with 9 follow-up study visits by phone or in person. The trial was registered through ClinicalTrials.gov (NCT04196140).

### 2.2. Participants

Two cohorts of participants were enrolled: children (6–13.9 years) and adolescents and adults (14–70 years); however, specific psychosocial measures were assessed according to the following group assignments: children (6–11.9 years), adolescents (12–17.9 years), and adults (18–70 years), with results for the adult group reported separately [13]. This report will focus on the 83 children and 42 adolescents who participated in the trial as well as the measures obtained from the caregivers of enrolled participants. Caregivers were considered an individual who is actively involved in the participant's diabetes management, with the same caregiver completing questionnaires at screening and the end of the study. The caregivers provided written informed consent, and children and adolescents provided assent.

### 2.3. Assessment of Psychosocial Outcomes

Participants and their caregivers completed questionnaires assessing diabetes distress (PAID), hypoglycemia confidence (HCS), mental well-being (WHO-5), sleep quality (PSQI), insulin delivery device satisfaction (IDSS), and system usability (SUS), as detailed below and summarized in Table S1. These patient-reported outcomes were chosen to assess a broad range of psychosocial outcomes in T1D management following use of this particular AID system. Questionnaires were completed before starting the AID system, in reference to the participants' standard therapy (“baseline”), and again based on the AID system following 3 months of use (“follow-up”). At follow-up, adolescents and caregivers of children also completed a free-response questionnaire about what they liked and disliked about the investigational system, and responses were summarized according to general themes. All questionnaires were completed online either in the clinic or at home.

### 2.4. Problem Areas in Diabetes

The Problem Areas in Diabetes (PAID) [14] questionnaire for children, adolescents, and their caregivers measures diabetes-specific emotional distress. Respondents rate how much each item applied to them over the last month on a six-point Likert scale from 1 = “Not a Problem” to 6 = “Big Problem”. Total scores are calculated as the sum of the questionnaire items. Versions of the PAID questionnaire include children (PAID-C) with 11 items and a possible score range of 11 to 66, caregivers of children (P-PAID-C) with 16 items and a possible score range of 16 to 96, adolescents (PAID-T) with 14 items and a possible score range of 14 to 84, and caregivers of adolescents (P-PAID-T) with 15 items and a possible score range of 15 to 90 [15, 16]. A lower score represents less distress.

### 2.5. Hypoglycemia Confidence Scale

The Hypoglycemia Confidence Scale (HCS) assesses the degree to which people with diabetes feel able, secure, and comfortable about their ability to avoid or address hypoglycemia-related problems, defined as confidence with hypoglycemia management [17]. Each of the 9 items on the HCS is rated on a 4-point scale from 1 (“Not Confident at All”) to 4 (“Very Confident”). A higher mean score indicates more confidence in managing hypoglycemia-related issues with a score ≥3 indicating relatively high confidence [17]. The HCS was completed by caregivers of children, adolescents, and caregivers of adolescents.

### 2.6. World Health Organization 5 Well-Being Index

The World Health Organization 5 Well-Being Index (WHO-5) measures overall well-being over the last two weeks and consists of 5 items rated on a 6-point (0 to 5) Likert scale with higher scores defining higher well-being [18]. Individual item scores are summed and multiplied by four to obtain a total percentage score with 0 representing the lowest possible well-being and 100 representing the best well-being. A percentage score ≤50 suggests low mood [19]. The WHO-5 was completed by caregivers of children, adolescents, and caregivers of adolescents.

### 2.7. Pittsburgh Sleep Quality Index

The Pittsburgh Sleep Quality Index (PSQI) measures sleep disturbance and typical sleep habits over the past month [20]. It differentiates poor sleep from good sleep by measuring 7 components: sleep quality, sleep latency, sleep duration, habitual sleep efficiency, sleep disturbances, use of sleep medication, and daytime dysfunction. The PSQI consists of 19 self-rated questions and 5 questions rated by the bed partner or roommate (if one was available). The 19 self-rated items are used to derive 7 component scores. These component scores range from 0 (better) to 3 (worse) and are then added together, resulting in a total score ranging from 0 (indicating no difficulty with sleep) to 21 (indicating severe difficulties with sleep). A total score ≥5 is considered an indicator of poor sleep quality. The PSQI was completed by adolescents and caregivers of children and adolescents.

### 2.8. Insulin Device Satisfaction Survey (T1)

The Insulin Device Satisfaction Survey (IDSS, T1 version) assesses patient satisfaction with their insulin delivery device along three dimensions: effectiveness, burden, and inconvenience [21]. The survey's 14 items are rated on a 5-point scale ranging from 1 (“Strongly Disagree”) to 5 (“Strongly Agree”). The three subscale scores are obtained by calculating the mean score for items associated with each subscale; the total score is based on the mean of all items with the responses to specific items for the burdensome and inconvenient subscales reverse-coded. For the overall scale and effectiveness subscale, a higher value indicates greater satisfaction, while for the burdensome and inconvenient subscales scores, a lower value defines greater satisfaction. The IDSS was completed by caregivers of children and caregivers of adolescents.

### 2.9. System Usability Scale

The System Usability Scale (SUS) is a validated questionnaire to assess perceived usability of technologies across industries [22, 23], including diabetes technology [24, 25]. The SUS is a 10-item questionnaire based on a 5-point Likert scale ranging from 1 (“Strongly Disagree”) to 5 (“Strongly Agree”). The items assess if users find the product intuitive and easy to use, how confident they feel about using the product, whether they think the product could be learned without technical support, and whether they think most people could learn to use it quickly. Answers are summed, with negatively worded questions reverse-coded, and then rescaled to give an overall usability index from 0 to 100. A higher score indicates a greater level of usability; a score of 68 is considered average. The SUS was completed by caregivers of children and by adolescents.

### 2.10. Statistical Methods

Psychosocial outcome scores were compared between baseline (in relation to participants' standard therapy) and after 3 months of AID system use, including total scores and subscales scores (if applicable). One participant in the children's cohort was withdrawn from the study due to an unrelated medical condition and did not complete follow-up questionnaires and was not included in the psychosocial outcome analysis. The distribution of change in each score was tested for normality using the Shapiro-Wilk test. For data that did not pass the test for normality or that had <10 participants with data, the Wilcoxon signed rank test was used. For normal data with >10 participants having data, the paired *t*-test was used. No adjustments for multiple comparisons were made. The magnitude of the treatment effect on change in score, i.e., the effect size, was calculated using Cohen's *d*, defined as the mean of the change divided by the standard deviation of the change [26]. A commonly used interpretation of Cohen's *d* is that the effect size is small (*d* = 0.2), medium (*d* = 0.5), or large (*d* = 0.8) [26]. Missing data were minimal and no meaningful differences existed between analyses results with or without data imputation for a majority of the questionnaires. The PSQI total score for caregivers of adolescents demonstrated slight differences with imputation; as sample size only differed by 3 participants (<10% missing data) with and without imputation, only the results of the initial analysis without imputation are presented.

Exploratory analyses using linear regression models were designed to assess the effects of various predictors on the change in psychosocial outcomes. For each questionnaire, a multiple linear regression model was used to assess the effects of 6 participant baseline factors (independent variables: baseline questionnaire score, age, diabetes duration, sex, percent time below range (TBR, % <70 mg/dL), and percent TIR (% 70–180 mg/dL)) on the dependent variable, namely change in individual psychosocial outcomes. Single linear regression models were used to assess the relationship between changes in glycemic outcomes, including both percent TBR and percent TIR, and changes in questionnaire scores. For consistency in interpreting the single and multiple linear regression results, the change in score for each questionnaire was calculated such that a positive change indicated an improvement. No adjustments for multiplicity were made to the models. All *P* values were considered significant by a two-sided value of 0.05. Analysis was performed using SAS version 9.4.

## 3. Results

### 3.1. Participant Characteristics

A total of 83 children and their caregivers and 42 adolescents and their caregivers, or dyads, were included in the study. Baseline characteristics of the children and adolescents are presented in Table 1. Twenty-three children (27.8%) were meeting the American Diabetes Association (ADA) target of HbA1c <7%, and 16 (19.3%) were meeting the consensus TIR target of >70% at baseline. Seven adolescents (16.7%) were meeting the ADA target of HbA1c <7% at baseline, and 6 (14.3%) were meeting the consensus TIR target at baseline. Ninety-nine percent of children (*n* = 82) and 100% percent of adolescents (*n* = 42) completed the full study.

### 3.2. Psychosocial Outcomes: Children and Their Caregivers

Psychosocial outcomes for children and their caregivers, including change from baseline, *P* values, and effect sizes, are summarized in Table 2. Significant improvements were observed in diabetes distress for children (PAID-C, *P* = 0.001) and their caregivers (P-PAID-C, *P* < 0.0001) after 3 months of AID use. Caregivers of children also had increased confidence in managing hypoglycemia (HCS, *P* < 0.0001) and improved general well-being (WHO-5, *P* = 0.03). While the PSQI total score did not improve, differences in the subscales for overall sleep quality (*P* < 0.0001), duration of sleep (*P* = 0.01), and sleep disturbance (*P* = 0.009), all indicated improvements. The four other PSQI subscales (sleep latency, day disturbance due to sleepiness, sleep efficiency, and needs medications to sleep) were not different following 3 months of AID use. Significant improvement in overall insulin delivery satisfaction (IDSS, *P* < 0.0001) and the IDSS subscales (effectiveness, *P* < 0.0001; burden, *P* = 0.0002; and inconvenience, *P* < 0.0001) as well as system usability (SUS, *P* < 0.0001) was observed following transition to the AID system.

An analysis of psychosocial outcomes stratified by prior standard therapy indicated some differences (Table S2). Caregivers of children previously using MDI or of those using tubed pumps saw greater improvement in insulin delivery satisfaction (IDSS) and system usability (SUS) than those previously on tubeless pump therapy (*P*=0.049 and *P* = 0.0009 for IDSS and *P* = 0.0002 and *P* = 0.002 for SUS, respectively). Further, caregivers of prior tubeless pump users had a greater improvement in sleep quality than caregivers of prior MDI users (*P* = 0.008) although only 3 caregivers of prior MDI users completed the PSQI questionnaire.

### 3.3. Psychosocial Outcomes: Adolescents and Their Caregivers

Psychosocial outcomes for adolescents and their caregivers, including change from baseline, *P* values, and effect sizes, are summarized in Table 3. Similar to children and their caregivers, adolescents and their caregivers experienced reduced diabetes distress (PAID-T, *P*=0.045 and P-PAID-T, *P* = 0.001, respectively). For caregivers of adolescents, overall satisfaction with the AID system (IDSS, *P* = 0.01) was greater compared with their previous treatment. Of the three IDSS subscales, caregivers reported significant reductions in perceived burden (*P* = 0.02) and inconvenience of insulin delivery (*P* = 0.04) but did not report a significant increase in perceived system effectiveness (*P* = 0.06). Additionally, system usability improved in adolescents (SUS, *P* = 0.002). Other measures (HCS, WHO-5, and PSQI-total) were not significantly different following 3 months of AID system use for either adolescents (*P* = 0.05, 0.06, and 0.17, respectively) or their caregivers (*P* = 0.18, 0.32, and 0.18, respectively). Furthermore, PSQI subscales were not significantly changed for either group following 3 months of AID system use (data not shown).

Adolescents using either MDI or tubed pumps for their standard therapy experienced greater improvement in system usability (SUS) than prior tubeless pump users by the end of the 3-month trial (*P*=0.02 and 0.005, respectively) (Table S3). Caregivers of adolescents transitioning from MDI also demonstrated greater improvements in overall insulin delivery satisfaction than caregivers of adolescents previously using tubeless pumps (*P* = 0.03). There were no significant differences between prior therapy methods for any of the other measures for adolescents or their caregivers.

### 3.4. Free Responses

Answers to the free response questionnaire about adolescents' and caregivers of children's likes and dislikes related to the system were categorized (Table 4). The most common (≥10% of respondents) positive themes included improved glucose management (30.1%), better nighttime control (22.0%), an appreciation of automated insulin delivery (16.3%), hypoglycemia prevention (11.4%), and reduced psychological burden (10.6%). When asked about what they liked most about the tubeless AID system, one caregiver said, “Now I barely think about diabetes…,” while another mentioned, “We didn't have to worry so much….” In contrast, commonly cited areas for improvement included pump failures (13.2%), alarm volume (10.7%), and algorithm performance (10.7%).

### 3.5. Regression Modelling

There were no significant baseline predictors (beyond baseline score), including glycemic outcomes, for changes in psychosocial outcomes scores for children, caregivers of children (Table S4), or adolescents (Table S5). However, among caregivers of adolescents, longer disease duration and higher % TBR at baseline were significant predictors of improvements in confidence with hypoglycemia management during system use (*P* = 0.048 and 0.02, respectively). Further, caregivers of adolescents who had higher % TBR at baseline demonstrated greater improvement in overall insulin delivery satisfaction (IDSS, *P* = 0.01). Finally, younger adolescent age was a significant predictor for improved sleep quality for these caregivers (PSQI, *P* = 0.04).

Single linear regression showed that, among children, the caregivers of children, and adolescents, there were no associations between any of the changes in glycemic outcomes and changes in psychosocial outcomes (Tables S4 and S5). For caregivers of adolescents, however, changes in questionnaire scores for two measures were correlated with changes in glycemic outcomes (Table S5): a greater decrease in % TBR was associated with a greater improvement confidence in managing hypoglycemia (HCS, *P* = 0.004) and a greater increase in % TIR was associated with a greater improvement in sleep quality (PSQI, *P* = 0.03).

## 4. Discussion

As a promising new technology, AID systems have the potential to alleviate the substantial and lifelong impact a diagnosis of T1D has on the physical health and psychological burden of children and adolescents, as well as their families. To our knowledge, this is the first report of psychosocial outcomes with a tubeless AID system in children, adolescents, and their caregivers. In this 3-month single-arm trial, all groups (children and adolescents with T1D, as well as their caregivers) using the Omnipod 5 AID System experienced decreases in diabetes-related distress. Caregivers of children also saw numerous additional benefits including increased confidence in managing hypoglycemia-related problems, increased well-being, increased insulin delivery satisfaction, and some measures of sleep quality, which occurred with a system that was perceived to be easier to use than the standard therapy utilized at baseline. There were fewer observed benefits in adolescents and their caregivers though adolescents reported greater device usability, and their caregivers reported increased insulin delivery satisfaction. In total, effect sizes for these outcomes are in the small/medium range, indicating clinically meaningful benefits.

In addition to improving glycemic outcomes, it is crucial that AID systems be easy to use without adding inconvenience or burden for the family [27]. Indeed, a systematic review focusing on the parental experience of caring for a child with T1D illustrated how diabetes can monopolize their lives [28]. Encouragingly, research has shown that people with diabetes and their families have high hopes for how AID systems may positively impact their lives [29, 30], and those using these systems in clinical trials have tended to show benefits in psychosocial outcomes such as diabetes distress [6, 7, 31, 32]. In the present study, the same trend was observed. Caregivers of children in particular were noted to experience the greatest number of measured benefits, including those reaching into areas beyond diabetes-related psychosocial outcomes: improved well-being and aspects of sleep. This result is supported by the recent finding that glycemia in children is associated with both the child's sleep quality as well as their parent's [33]. Contrary to caregivers of children, caregivers of adolescents did not experience an improvement in well-being or sleep quality, yet younger age was found to be a predictor of improved sleep quality for this group. Given that as the adolescent ages, there is a transition of responsibility for diabetes management from the caregiver to the adolescent [34], this result is not surprising since younger adolescents are less likely to be independent in their overnight care. Notably, an increase in TIR and a reduction in TBR for adolescents were associated with improved sleep quality and confidence in managing hypoglycemia for their caregivers, respectively. These findings may be explained by previous studies which have highlighted the relationship between impaired glycemia in adolescents with T1D and higher levels of parental stress and frequent disagreements on diabetes-related family responsibilities [35, 36]. These results extend prior findings of improved glycemic outcomes associated with the Omnipod 5 AID System [10, 11] and underscore the potential for families to not only improve their child's physical health but also reduce the psychological burden experienced by all family members.

Among other AID studies in children and adolescent age groups, diabetes distress and sleep quality are the two measures that have been most consistently measured [5–7, 32, 37–39], with a few studies assessing these measures specifically among younger children and their caregivers. In a nonrandomized clinical trial of 13 children (7–10 y) using AID, parents saw significant improvements in diabetes distress following one month of AID use, with parents also reporting improved sleep quality [4]. In a longer 28-week crossover randomized controlled trial (RCT) of 101 children (6–13 y) using the same AID system, parents of children using AID experienced significant improvements in diabetes distress and sleep quality, with 27 out of 49 poor sleepers becoming good sleepers [5, 38]. Yet, in the same study, children using AID did not experience improvements in diabetes distress although benefits were observed in other areas that were not assessed in the present study (quality of life, fear of hypoglycemia) [37].

Additionally, several studies have focused on the impact specifically for the adolescent age group. In a 4-month crossover study of 24 adolescents (10–18 y) initiating AID use, adolescents reported significantly improved diabetes distress [31]. Similarly, a real-world study of 115 adolescents aged 11–18 y with T1D and 243 parents of children aged <18 y initiating an AID system reported improved sleep quality in both adolescents and parents following 6 months of use [7]. However, a 3-month AID trial among 37 adolescents (10–17 y) and their caregivers found no significant changes in diabetes distress or sleep quality [6]. Additionally, in a RCT of another AID system that included both children and adolescents (6–18 y) and their caregivers, 6 months of system use was not associated with psychosocial benefits overall although there were modest improvements in quality of life and parent distress in a small group using a specific version of the system [32].

Thus, while all other AID studies seem to demonstrate improved glycemic control, to date, the added benefits of significant improvements in sleep quality and diabetes distress for children, adolescents, and their caregivers vary between systems and studies. Potential reasons for these variations could include glycemia and psychosocial status at baseline, patient comfort in adoption of new diabetes technology, extent of caregiver involvement in their child's diabetes management, and study design (e.g., observational vs. RCT, duration of study), in addition to characteristics of the individual AID systems evaluated. Future studies should continue to evaluate these psychosocial outcomes as critical measures of success for AID systems to provide a more complete picture of the benefits these systems can have for users and their families.

Strengths of this study include a relatively wide range of both children and adolescent participants with respect to prior insulin delivery modality including MDI, insulin pump, and AID users. The study also incorporated a broad range of psychosocial measures completed by both participants and caregivers to obtain a more complete understanding of the various aspects of psychosocial outcomes with tubeless AID. While many questionnaires were used, there was a high participation rate for children and adolescents in the main safety and efficacy study as demonstrated in Tables 2 and 3. Another strength of this study includes the focus on separate analyses of children-caregiver and adolescent-caregiver dyads, which is limited in the literature. Additionally, inclusion of free response questions provided more descriptive assessment regarding use of the system and provided additional details regarding the participants' lived experiences not captured in standardized instruments.

Limitations for this study include a single-arm design without a control group comparison and psychosocial outcomes were not included as endpoints in the clinical study protocol; thus, we cannot be certain that the observed improvements in the various psychosocial outcomes were the sole result of the intervention. Secondly, the study was limited to a 3-month period with frequent follow-up visits during the study. Thus, with the present data, it is uncertain whether the frequency of visits impacted the observed psychosocial benefits and whether these benefits will be maintained over time. Furthermore, the results for subgroups of prior MDI or tubed pump therapy users should be interpreted with caution due to the small sample size. Another limitation of the current study is that the majority of study participants (84.3% of children and 92.9% of adolescents) were non-Hispanic white, thus limiting our understanding of psychosocial outcomes across diverse racial and ethnic groups. Additionally, income and education level of participants were not collected and are unknown for the study population. Future studies will include a postmarket study to collect this type of patient information and evaluate the system in a larger sample that is more representative of the overall T1D population. Within these constraints, however, we conclude that use of the Omnipod 5 AID System was associated with significant improvements in psychosocial outcomes in this population of children and adolescents with T1D and their caregivers.

## 5. Conclusion

Children and adolescents with T1D and their families face numerous challenges to diabetes management due to the considerable demands of the condition. To determine how technology might alleviate some portion of these burdens, this study assessed a range of key psychosocial outcomes among children, adolescents, and their caregivers transitioning to a tubeless AID system. The results demonstrated that introduction of the Omnipod 5 AID System was associated with beneficial reductions in diabetes distress for all groups assessed, with additional benefits noted among caregivers of children. Taken together, these findings support the broad impact that AID systems could offer to potentially reduce many of the emotionally and psychologically taxing aspects of managing diabetes for the entire family.

## Figures and Tables

**Table 1:** Characteristics of participants at baseline.

Characteristics	Children (6–11.9 years)	Adolescents (12–17.9 years)
*N*	83^†^	42
Age (years), [range]^‡^	9.4 ± 1.7 [6.0, 12.0]	14.0 ± 1.7 [12.0, 18.0]
Duration of diabetes (years)	4.3 ± 2.3 [0.6, 10.7]	6.7 ± 3.5 [1.1, 13.8]
Body-mass index (kg/m^2^), [range]^§^	17.8 ± 2.5 [13.7, 31.6]	21.4 ± 3.7 [16.0, 32.4]
Female sex-no. (%)	44 (53.0)	22 (52.4)
Race/Ethnicity-no. (%)^¶^		
White	78 (94.0)	39 (92.9)
Hispanic or latino	8 (9.6)	0 (0.0)
Not hispanic or latino	70 (84.3)	39 (92.9)
Black or African American, white	2 (2.4)	1 (2.4)
Black or African American	2 (2.4)	—
Asian, white	1 (1.2)	1 (2.4)
Asian, native Hawaiian or pacific islander, white	—	1 (2.4)
Time in range (TIR) 70–180 mg/dL (%)	55.1 ± 14.8	49.6 ± 16.2
TIR >70%-no. (%)	16 (19.3)	6 (14.3)
HbA1c (%), [range]^††^	7.5 ± 0.9 [5.8, 9.8]	7.9 ± 1.0 [5.8, 10.3]
HbA1c (mmol/mol), [range]^††^	58 ± 9.8 [40, 84]	63 ± 10.9 [40, 89]
HbA1c <7.0%-no. (%)	23 (27.8)	7 (16.7)
Previous^‡‡^ or current continuous glucose monitor use-no. (%)	82 (98.8)	39 (92.9)
Baseline standard therapy, MDI-no. (%)	8 (9.6)	7 (16.7)
Baseline standard therapy, pump-no. (%)	75 (90.4)	35 (83.3)
Tubeless pump	60 (72.3)	28 (66.7)
Tubed pump		
With low glucose suspend capabilities	2 (2.4)	2 (4.8)
With predictive low glucose suspend capabilities	2 (2.4)	1 (2.4)
With hybrid closed-loop capabilities	7 (8.4)	4 (9.5)

Data are mean ± SD and range [minimum, maximum] or *n* (%). ^†^One participant was removed from further psychosocial outcomes analysis due to a medical condition that removed them from the study. ^‡^Age was determined at the date of informed consent. ^§^Body-mass index is the weight in kilograms divided by the square of the height in meters. ^¶^Race and ethnicity were reported by the participants and were not mutually exclusive. ^††^Participant eligibility for the study was determined using a point-of-care HbA1c measurement performed at screening, which in some cases differed from the laboratory assessment displayed here and used for analysis. ^‡‡^Previous use is defined as having used the device for any duration in the past.

**Table 2:** Questionnaire and subscale scores between children and their caregivers (*N* = 82) at baseline and after 3 months of AID use.

Questionnaire	*N*	Score range	Baseline	3 months of AID	Change^†^	*P* value	Cohen's *d*
*Children*							
PAID-C	75	11 to 66^‡^	27.4 ± 9.8	24.2 ± 9.3	−3.2 ± 8.1	**0.0010** ^1^	0.39
			26.0 [20.0, 35.0]	23.0 [17.0, 31.0]	−3.0 [−9.0, 0.0]		

*Caregivers of children*							
P-PAID-C	76	16 to 96^‡^	47.1 ± 14.3	40.7 ± 10.8	−6.3 ± 12.2	**<0.0001** ^1^	0.52
			48.5 [37.0, 55.0]	42.5 [32.0, 48.0]	−6.0 [−14.0, −0.5]		
HCS	81	1 to 4	3.34 ± 0.48	3.59 ± 0.39	0.24 ± 0.56	**<0.0001** ^2^	0.43
			3.33 [3.00, 3.75]	3.67 [3.44, 3.89]	0.22 [0.00, 0.67]		
WHO-5	81	0 to 100	67.9 ± 17.2	72.8 ± 13.4	5.0 ± 18.3	**0.0331** ^2^	0.27
			72.0 [56.0, 80.0]	76.0 [64.0, 80.0]	4.0 [−8.0, 12.0]		
PSQI-total	61	0 to 21^‡^	6.54 ± 3.91	5.77 ± 2.75	−0.77 ± 3.73	0.1123^1^	0.21
			6.00 [3.00, 9.00]	6.00 [3.00, 8.00]	0.00 [−3.00, 1.00]		
Duration of sleep	81	0 to 3^‡^	0.63 ± 0.93	0.35 ± 0.74	−0.28 ± 1.00	**0.0099** ^2^	0.28
Sleep disturbance	74	0 to 3^‡^	1.26 ± 0.50	1.08 ± 0.46	−0.18 ± 0.58	**0.0091** ^2^	0.30
Overall sleep quality	82	0 to 3^‡^	1.12 ± 0.84	0.70 ± 0.66	−0.43 ± 0.83	**<0.0001** ^2^	0.51
IDSS-Overall	82	1 to 5	3.79 ± 0.56	4.13 ± 0.60	0.34 ± 0.66	**<0.0001** ^1^	0.53
			3.82 [3.43, 4.14]	4.25 [3.79, 4.57]	0.36 [−0.14, 0.71]		
Effective	82	1 to 5	4.10 ± 0.55	4.38 ± 0.65	0.27 ± 0.74	**<0.0001** ^2^	0.37
Burdensome	82	1 to 5^‡^	2.30 ± 0.70	1.96 ± 0.70	−0.34 ± 0.80	**0.0002** ^1^	0.43
Inconvenient	82	1 to 5^‡^	2.50 ± 0.81	2.06 ± 0.79	−0.44 ± 0.91	**<0.0001** ^1^	0.48
SUS	78	0 to 100	77.5 ± 16.8	89.0 ± 12.1	11.5 ± 19.1	**<0.0001** ^2^	0.60
			77.5 [70.0, 90.0]	95.0 [82.5, 97.5]	10.0 [0.0, 22.5]		

Data are mean ± standard deviation. Unless otherwise indicated, remaining values are median [IQR]. IQR denotes interquartile range. ^†^Change is calculated as follow-up after 3 months of AID minus baseline score. ^‡^Lower value indicates optimal score. ^1^Unadjusted two-sided*P* value for paired *t*-test. ^2^Unadjusted two-sided Wilcoxon signed rank test. Bolded values represent *p* values that were statistically significant.

**Table 3:** Questionnaire and subscale scores between adolescents and their caregivers (*N* = 42) at baseline and after 3 months of AID use.

Questionnaire	*N*	Score range	Baseline	3 months of AID	Change^†^	*P* value	Cohen's *d*
*Adolescents*							
PAID-T	38	14 to 84^‡^	30.5 ± 11.4	27.1 ± 10.8	−3.4 ± 10.2	**0.0451** ^1^	0.34
			28.5 [21.0, 38.0]	25.5 [19.0, 32.0]	−2.0 [−10.0, 3.0]		
HCS	42	1 to 4	3.41 ± 0.42	3.54 ± 0.42	0.13 ± 0.42	0.0525^1^	0.31
			3.35 [3.00, 3.86]	3.63 [3.25, 3.88]	0.09 [−0.13, 0.38]		
WHO-5	40	0 to 100	71.0 ± 14.7	74.2 ± 16.5	3.2 ± 11.9	0.0595^2^	0.27
			72.0 [64.0, 78.0]	76.0 [66.0, 84.0]	4.0 [0.0, 8.0]		
PSQI-total	25	0 to 21^‡^	5.08 ± 2.48	4.36 ± 2.55	−0.72 ± 2.56	0.1721^1^	0.28
			5.00 [4.00, 6.00]	4.00 [2.00, 6.00]	0.00 [−2.00, 1.00]		
SUS	40	0 to 100	79.4 ± 16.2	86.3 ± 20.1	6.9 ± 23.7	**0.0015** ^2^	0.29
			80.0 [70.0, 93.8]	95.0 [81.3, 98.8]	5.0 [0.0, 20.0]		

*Caregivers of adolescents*							
P-PAID-T	35	15 to 90^‡^	45.0 ± 14.2	38.0 ± 13.8	−7.0 ± 11.9	**0.0014** ^1^	0.59
			45.0 [34.0, 56.0]	38.0 [28.0, 48.0]	−7.0 [−14.0, 3.0]		
HCS	42	1 to 4	3.31 ± 0.46	3.43 ± 0.41	0.12 ± 0.55	0.1761^1^	0.21
			3.25 [3.00, 3.67]	3.47 [3.11, 3.78]	0.05 [−0.11, 0.45]		
WHO-5	41	0 to 100	65.2 ± 12.3	67.8 ± 13.9	2.6 ± 16.6	0.3158^1^	0.16
			64.0 [56.0, 76.0]	68.0 [60.0, 80.0]	4.0 [−4.0, 16.0]		
PSQI-total	35	0 to 21^‡^	6.00 ± 2.61	5.31 ± 2.69	−0.69 ± 2.71	0.1848^2^	0.25
			6.00 [4.00, 8.00]	4.00 [3.00, 7.00]	0.00 [−2.00, 1.00]		
IDSS-overall	42	1 to 5	3.82 ± 0.65	4.12 ± 0.46	0.30 ± 0.75	**0.0142** ^1^	0.40
			3.86 [3.50, 4.21]	4.18 [3.71, 4.50]	0.32 [−0.21, 0.64]		
Effective	41	1 to 5	4.09 ± 0.61	4.36 ± 0.65	0.27 ± 0.89	0.0583^1^	0.30
Burdensome	41	1 to 5^‡^	2.21 ± 0.74	1.94 ± 0.47	−0.27 ± 0.76	**0.0241** ^2^	0.36
Inconvenient	42	1 to 5^‡^	2.49 ± 0.86	2.15 ± 0.64	−0.34 ± 1.02	**0.0358** ^1^	0.34

Data are mean ± standard deviation. Unless otherwise indicated, remaining values are median [IQR]. IQR denotes interquartile range. ^†^Change is calculated as follow-up after 3 months of AID minus baseline score. ^‡^Lower value indicates optimal score. ^1^Unadjusted two-sided*P* value for paired *t*-test. ^2^Unadjusted two-sided Wilcoxon signed rank test.

**Table 4:** Categorization of free response answers to questionnaire on likes and dislikes of the tubeless AID system between adolescents and caregivers of children, showing the most commonly reported response categories for each question (reported by ≥10% of participants).

Answer category	Number of users^†^ (%)	Representative quote
*1. What did you like most about [the study system]? (n* *=* *123*^*†*^)		
Improved BG management	37 (30.1)	I liked most about the [system] is that it kept [my child's] blood glucose levels more in target range. It seems like his blood glucose is more in range especially during the night and when waking up in the morning. I have also notice[d] that he doesn't need to treat lows as often and needs snacks as often because his blood glucose is more steady and well controlled with the AID. I also like that myself and [my child] are not always thinking about diabetes all the time and it gives us a break from it. -Caregiver of a child
Night time control/better sleep	27 (22.0)	Helped reduce volatility, especially at night. It used to be an exception to have a good night sleep and not having to go manage our child's T1D, with [the system] it is an exception that we have to go out of bed to manage our child's T1D. -Caregiver of a child
Automated insulin delivery	20 (16.3)	What I liked the most about the [system] is the automated system. We didn't have to worry so much about correcting and bolusing especially overnight. I can say, I (mom) have slept with no worries and in peace these past few months since using the […] system. It has been a blessing!!!!! -Caregiver of a child
Prevented lows	14 (11.4)	I Like that it helped prevent low blood sugars because I didn't have to stop and treat my lows as much. -Teen
Reduced psychological burden	13 (10.6)	My child is extremely insulin and carb sensitive. He was on diluted insulin until nearly age 7, and required extended boluses for nearly every food. I was diligent and worked hard, and yet his A1c prior to the start of the trial was nearly 9.0. Now I barely think about diabetes, his A1c is at or below target, and he rarely has low blood sugar. Couldn't be happier and can't wait for commercial approval. -Caregiver of a child

*2. What did you like least about [the system]? (n* *=* *121*^*†*^)		
Pod failures	16 (13.2)	Pods tended to have errors [⋯] resulting in the need to change more often than every 3 days. -Caregiver of a child
Liked everything	15 (12.4)	I don't have anything negative to say about the [system]. -Teen
Alarm volume	13 (10.7)	Mostly the sound effects and alerts I couldn't mute. -Teen
Algorithm performance	13 (10.7)	It isn't overly aggressive and needs to be more aggressive to hit the target bg. -Teen

^†^123 and 121 participants responded to these questions, respectively (out of 125 total participants); however, only response categories reported by ≥10% of participants are shown. There were additional response categories reported by <10% of participants, which are not shown.

## Data Availability

The datasets generated during and/or analyzed during the current study are not publicly available.

## Supplementary Materials

Supplementary Materials Table S1: list of questionnaires for assessment of psychosocial outcomes by participant group. Table S2: comparison of differences in psychosocial outcomes for children and their caregivers between prior standard therapies (MDI, tubed pump, or tubeless pump). Table S3: comparison of differences in psychosocial outcomes for adolescents and their caregivers between prior standard therapies (MDI, tubed pump, or tubeless pump). Table S4: estimated coefficients for multiple and single linear regression of change in questionnaire score among children and their caregivers. Table S5: estimated coefficients for multiple and single linear regression of change in questionnaire score among adolescents and their caregivers.
