# Supplementary material for: Psychosocial Outcomes with the Omnipod® 5 Automated Insulin Delivery System in Children and Adolescents with Type 1 Diabetes and Their Caregivers
Source: Pediatr Diabetes. 2023 Jun 29;2023:8867625. doi: 10.1155/2023/8867625 (PMC12017088; doi:10.1155/2023/8867625)
Supplement: Supplementary Materials — Table S1: list of questionnaires for assessment of psychosocial outcomes by participant group. Table S2: comparison of differences in psychosocial outcomes for children and their caregivers between prior standard therapies (MDI, tubed pump, or tubeless pump). Table S3: comparison of differences in psychosocial outcomes for adolescents and their caregivers between prior standard therapies (MDI, tubed pump, or tubeless pump). Table S4: estimated coefficients for multiple and single linear regression of change in questionnaire score among children and their caregivers. Table S5: estimated coefficients for multiple and single linear regression of change in questionnaire score among adolescents and their caregivers. [file 8867625.f1.pdf]

Title:

Psychosocial Outcomes with the Omnipod® 5 Automated Insulin Delivery System in Children and Adolescents with Type 1 Diabetes and their Caregivers

Authors:

Korey K. Hood PhD[1], William H. Polonsky PhD, CDCES[2,3], Sarah A. MacLeish DO[4], Carol J. Levy MD, CDCES[5], Gregory P. Forlenza MD MS[6], Amy B. Criego MD MS[7], Bruce A. Buckingham MD[8], Bruce W. Bode MD[9], David W. Hansen MD, MPH[10], Jennifer L. Sherr MD, PhD[11], Sue A. Brown MD[12], Daniel J. DeSalvo MD[13], Sanjeev N. Mehta MD, MPH[14], Lori M. Laffel MD, MPH[14], Anuj Bhargava MD, MBA, CDCES, FACP, FACE,[15] Lauren M. Huyett PhD[16], Todd E. Vienneau BSc[16], Trang T. Ly MBBS, FRACP, PhD[16] for the Omnipod 5 Research Group

Affiliations:

[1] Department of Pediatrics, Psychiatry & Behavioral Sciences, Stanford Diabetes Research Center, Stanford University School of Medicine, Stanford University, Stanford, CA

[2] University of California San Diego, San Diego, CA

[3] Behavioral Diabetes Institute, San Diego, CA

[4] University Hospitals Cleveland Medical Center, Rainbow Babies and Children's Hospital, Cleveland, OH

[5] Icahn School of Medicine at Mount Sinai, New York, NY

[6] Barbara Davis Center for Diabetes, University of Colorado Anschutz Medical Campus, Aurora, CO

[7] International Diabetes Center, Health Partners Institute, Minneapolis, MN

[8] Department of Pediatrics, Division of Pediatric Endocrinology, Stanford University, Stanford, CA

[9] Atlanta Diabetes Associates, Atlanta, GA

[10] Department of Pediatrics, SUNY Upstate Medical University, Syracuse, NY

[11] Department of Pediatrics, Yale School of Medicine, New Haven, CT

[12] Division of Endocrinology, Center for Diabetes Technology, University of Virginia, Charlottesville, VA

[13] Department of Pediatrics, Baylor College of Medicine, Houston, TX

[14] Joslin Diabetes Center, Harvard Medical School, Boston, MA

[15] Department of Research, Iowa Diabetes Research, West Des Moines, IA

[16] Insulet Corporation, Acton, MA

Corresponding author:

Trang T. Ly

100 Nagog Park, Acton, MA 01720

phone: 978-600-7628

email: tly@insulet.com

SUPPLEMENTARY MATERIAL

This supplementary material has been provided by the authors to give readers additional information about their work.

Supplement to: Hood et al. Psychosocial Outcomes with the Omnipod® 5 Automated Insulin Delivery System in Children and Adolescents with Type 1 Diabetes and their Caregivers

Contents

Table S1. List of questionnaires for assessment of psychosocial outcomes by participant group 4

Table S2. Comparison of differences in psychosocial outcomes for children and their caregivers between prior standard therapies (MDI, tubed pump, or tubeless pump).. 5

Table S3. Comparison of differences in psychosocial outcomes for adolescents and their caregivers between prior standard therapies (MDI, tubed pump, or tubeless pump)... 6

Table S4. Estimated coefficients for multiple and single linear regression of change in questionnaire score among children and their caregivers† 7

Table S5. Estimated coefficients for multiple and single linear regression of change in questionnaire score among adolescents and their caregivers†... 8

Table S1. List of questionnaires for assessment of psychosocial outcomes by participant group

Questionnaire	Children	Caregivers of Children	Adolescents	Caregivers of Adolescents
**PAID-C**	X			
**P-PAID-C**		X		
**PAID-T**			X	
**P-PAID-T**				X
**HCS**		X	X	X
**WHO-5**		X	X	X
**PSQI**		X	X	X
**IDSS**		X		X
**SUS**		X	X	

Abbreviations: PAID-C, Problem Areas in Diabetes – Child; P-PAID-C, Parent – Problem Areas in Diabetes – Child; PAID-T, Problem Areas in Diabetes – Teen; P-PAID-T, Parent – Problem Areas in Diabetes – Teen; HCS, Hypoglycemia Confidence Scale; WHO-5, World Health Organization Well-Being Index 5; PSQI, Pittsburgh Sleep Quality Index; IDSS, Insulin Delivery System Satisfaction; SUS, System Usability Scale

Table S2. Comparison of differences in psychosocial outcomes for children and their caregivers between prior standard therapies (MDI, tubed pump, or tubeless pump)

Questionnaire	MDI		Tubed		Tubeless		Mean Difference ± SEM (Tubeless - MDI)	P-value‡	Mean Difference ± SEM (Tubed - MDI)	P-value‡	Mean Difference ± SEM (Tubed - Tubeless)	P-value‡
	N	Mean Change†	N	Mean Change†	N	Mean Change†						
**Children**												
**PAID-C**	7	-0.4	15	-1.5	53	**-4.1*** **	-3.6 ± 3.5	0.3024	-1.0 ± 4.1	0.8007	2.6 ± 2.1	0.2320
**Caregivers of Children**												
**P-PAID-C**	7	-10.6	14	**-8.9****	55	**-5.2****	5.4 ± 5.1	0.2910	1.7 ± 5.1	0.7394	-3.7 ± 3.6	0.3123
**HCS**	8	0.28	15	0.26	58	**0.23*****	-0.05 ± 0.22	0.8111	-0.02 ± 0.20	0.9145	0.03 ± 0.17	0.8521
**WHO-5**	8	10.0	14	-3.1	59	**6.2***	-3.8 ± 7.0	0.5938	-13.1 ± 8.7	0.1476	-9.4 ± 5.1	0.0702
**PSQI**	3	4.67	11	0.45	47	**-1.40****	-6.07 ± 2.20	**0.0081**	-4.21 ± 2.09	0.0667	1.86 ± 1.14	0.1084
**IDSS**	8	0.64	15	**0.79*****	59	**0.19***	-0.45 ± 0.23	**0.0494**	0.15 ± 0.31	0.6461	0.60 ± 0.17	**0.0009**
**SUS**	7	**31.4***	14	**22.5****	57	**6.3****	-25.1 ± 6.3	**0.0002**	-8.9 ± 9.9	0.3790	16.2 ± 5.1	**0.0021**

*p<0.05, **p<0.01, ***p<0.001

† Change is calculated as follow-up after 3 months of AID minus baseline score

‡ Difference between indicated groups by two-sided t-test

Abbreviations: MDI, multiple daily injections; SEM, standard error of the mean; PAID-C, Problem Areas in Diabetes – Child; P-PAID-C, Parent – Problem Areas in Diabetes – Child; HCS, Hypoglycemia Confidence Scale; WHO-5, World Health Organization Well-Being Index 5; PSQI, Pittsburgh Sleep Quality Index; IDSS, Insulin Delivery System Satisfaction; SUS, System Usability Scale

Table S3. Comparison of differences in psychosocial outcomes for adolescents and their caregivers between prior standard therapies (MDI, tubed pump, or tubeless pump)

	MDI		Tubed		Tubeless		Mean Difference ± SEM (Tubeless - MDI)	P-value‡	Mean Difference ± SEM (Tubed - MDI)	P-value‡	Mean Difference ± SEM (Tubed - Tubeless)	P-value‡
**Questionnaire**	**N**	**Mean Change†**	**N**	**Mean Change†**	**N**	**Mean Change†**						
**Adolescents**												
**PAID-T**	7	0.0	7	-3.7	24	-4.3	-4.3 ± 4.6	0.3522	-3.7 ± 4.9	0.4607	0.6 ± 4.5	0.8910
**HCS**	7	0.13	7	0.38	28	0.07	-0.07 ± 0.17	0.6974	0.25 ± 0.30	0.4258	0.31 ± 0.16	0.0534
**WHO-5**	7	0.6	6	2.7	27	**4.0***	3.4 ± 5.0	0.4970	2.1 ± 9.4	0.8277	-1.3 ± 4.6	0.7750
**PSQI**	5	-1.80	6	0.17	14	-0.71	1.09 ± 1.49	0.4771	1.97 ± 1.39	0.1916	0.88 ± 1.18	0.4658
**SUS**	6	**22.1***	7	26.4	27	-1.5	-23.6 ± 9.2	**0.0151**	4.3 ± 10.3	0.6811	27.9 ± 9.3	**0.0052**
**Caregivers of Adolescents**												
**P-PAID-T**	7	0.0	5	-12.2	23	**-8.0****	-8.0 ± 4.5	0.0852	-12.2 ± 7.8	0.1501	-4.2 ± 6.0	0.4889
**HCS**	7	0.16	7	0.43	28	0.03	-0.13 ± 0.23	0.5873	0.28 ± 0.28	0.3386	0.40 ± 0.24	0.1006
**WHO-5**	7	-3.4	7	2.9	27	4.1	7.6 ± 7.2	0.3011	6.3 ± 8.1	0.4528	-1.3 ± 7.3	0.8603
**PSQI**	7	0.43	7	-2.29	21	-0.52	-0.95 ± 1.02	0.3606	-2.71 ± 1.49	0.0934	-1.76 ± 1.24	0.1660
**IDSS**	7	0.88	7	0.16	28	0.19	-0.69 ± 0.31	**0.0328**	-0.72 ± 0.52	0.1940	-0.02 ± 0.25	0.9264

*p<0.05, **p<0.01, ***p<0.001

† Change is calculated as follow-up after 3 months of AID minus baseline score

‡ Difference between indicated groups by two-sided t-test

Abbreviations: MDI, multiple daily injections; SEM, standard error of the mean; PAID-T, Problem Areas in Diabetes – Teen; P-PAID-T, Parent – Problem Areas in Diabetes – Teen; HCS, Hypoglycemia Confidence Scale; WHO-5, World Health Organization Well-Being Index 5; PSQI, Pittsburgh Sleep Quality Index; IDSS, Insulin Delivery System Satisfaction; SUS, System Usability Scale

Table S4. Estimated coefficients for multiple and single linear regression of change in questionnaire score among children and their caregivers[†]

	Multiple Linear Regression						Single Linear Regression	
	Baseline Score	Age (yrs)	Diabetes Duration (yrs)	Sex[‡]	Baseline/ST % TBR <70 mg/dL	Baseline/ST % TIR 70-180 mg/dL	Δ % TBR <70 mg/dL[§]	Δ % TIR 70-180 mg/dL[¶]
**Children**								
**ΔPAID-C**	**0.449********	-0.051	-0.567	-1.095	-0.010	0.096	-0.101	-0.060
**Caregivers of Children**								
**ΔP-PAID-C**	**0.573******	0.030	-0.708	-0.084	0.483	-0.020	-1.241	0.024
**ΔHCS**	**-0.858******	0.005	-0.016	0.034	-0.016	0.004	-0.023	-0.007
**ΔWHO-5**	**-0.765******	0.942	-1.049	-0.385	-0.671	0.131	-0.038	-0.377
**ΔPSQI** - Total	**0.712******	0.175	-0.307	-1.131	0.108	0.016	-0.037	-0.063
**ΔIDSS** - Overall	**-0.615******	-0.003	0.002	-0.069	-0.040	0.003	0.060	0.003
**ΔSUS**	**-0.889******	1.305	-1.221	-3.743	-0.183	0.049	0.801	-0.094

Data are estimated coefficient (β), *p<0.05, **p<0.01, ***p<0.001

[†] For consistency in interpreting the regression analysis, the change for each questionnaire was calculated in such a way that a positive change indicates an improvement: baseline subtracted from follow-up for questionnaires where a higher score represents an improvement (HCS, WHO-5, IDSS, SUS), and follow-up subtracted from baseline for questionnaires where a lower score represents an improvement (PAID, PSQI).

[‡] Sex was coded such that male was set to 0 and female was set to 1.

[§] Change in percent TBR is calculated as follow-up minus baseline. A negative coefficient means that a larger (more negative) decrease in TBR was associated with a greater improvement in the questionnaire score outcome.

[¶] Change in percent TIR is calculated as follow-up minus baseline. A positive coefficient means that a greater increase in TIR was associated with a greater improvement in the questionnaire score outcome.

Abbreviations: ST, standard therapy; TBR, time below range; TIR, time in range; PAID-C, Problem Areas in Diabetes – Child; P-PAID-C, Parent – Problem Areas in Diabetes – Child; HCS, Hypoglycemia Confidence Scale; WHO-5, World Health Organization Well-Being Index 5; PSQI, Pittsburgh Sleep Quality Index; IDSS, Insulin Delivery System Satisfaction; SUS, System Usability Scale

Table S5. Estimated coefficients for multiple and single linear regression of change in questionnaire score among adolescents and their caregivers[†]

	Multiple Linear Regression						Single Linear Regression	
	Baseline Score	Age (yrs)	Diabetes Duration (yrs)	Sex[‡]	Baseline/ST % TBR <70 mg/dL	Baseline/ST % TIR 70-180 mg/dL	Δ % TBR <70 mg/dL[§]	Δ % TIR 70-180 mg/dL[¶]
**Adolescents**								
**ΔPAID-T**	**0.459****	-1.212	0.887	-1.956	0.425	-0.031	-0.425	0.107
**ΔHCS**	**-0.555*****	-0.013	0.032	0.016	0.012	0.002	-0.008	0.002
**ΔWHO-5**	-0.240	-1.069	0.596	-4.135	0.266	0.019	0.396	0.015
**ΔPSQI** - Total	0.393	0.043	-0.118	-1.699	-0.010	0.027	0.234	-0.031
**ΔSUS**	**-0.850*****	-0.752	-0.926	-5.217	1.528	-0.142	-0.560	0.024
**Caregivers of Adolescents**								
**ΔP-PAID-T**	**0.362***	-0.285	0.687	-0.156	0.463	-0.046	-0.733	0.050
**ΔHCS**	**-0.685*****	-0.046	**0.039***	0.107	**0.055***	-0.005	**-0.108****	0.006
**ΔWHO-5**	**-0.709****	-1.219	0.290	4.567	0.169	-0.073	0.234	0.312
**ΔPSQI** - Total	**0.576****	**-0.520***	0.017	-1.085	0.007	-0.044	0.135	**0.077***
**ΔIDSS** - Overall	**-0.955*****	-0.016	-0.004	-0.063	**0.074***	-0.006	-0.060	0.001

Data are estimated coefficient (β), *p<0.05, **p<0.01, ***p<0.001

[†] For consistency in interpreting the regression analysis, the change for each questionnaire was calculated in such a way that a positive change indicates an improvement: baseline subtracted from follow-up for questionnaires where a higher score represents an improvement (HCS, WHO-5, IDSS, SUS), and follow-up subtracted from baseline for questionnaires where a lower score represents an improvement (PAID, PSQI).

[‡] Sex was coded such that male was set to 0 and female was set to 1.

[§] Change in percent TBR is calculated as follow-up minus baseline. A negative coefficient means that a larger (more negative) decrease in TBR was associated with a greater improvement in the questionnaire score outcome.

[¶] Change in percent TIR is calculated as follow-up minus baseline. A positive coefficient means that a greater increase in TIR was associated with a greater improvement in the questionnaire score outcome.

Abbreviations: ST, standard therapy; TBR, time below range; TIR, time in range; PAID-T, Problem Areas in Diabetes – Teen; P-PAID-T, Parent – Problem Areas in Diabetes – Teen; HCS, Hypoglycemia Confidence Scale; WHO-5, World Health Organization Well-Being Index 5; PSQI, Pittsburgh Sleep Quality Index; IDSS, Insulin Delivery System Satisfaction; SUS, System Usability Scale
